# Comparison of HLA-A, -B and -DRB1 Loci Polymorphism between Kidney Transplants of Uremia Patients and Healthy Individuals in Central China

**DOI:** 10.1371/journal.pone.0165426

**Published:** 2016-10-25

**Authors:** Wenjun Shang, Yuefeng Shen, Shilin Gao, Guiwen Feng, Yonghua Feng, Zhigang Wang, Xiaobai Zhang

**Affiliations:** 1 The Department of Kidney Transplantation, the First Affiliated Hospital of Zhengzhou University, Zhengzhou, Henan, China; 2 Shanghai Key Laboratory of Signaling and Disease Research, the School of Life Sciences and Technology, Tongji University, Shanghai, China; Jilin University, CHINA

## Abstract

Chronic kidney disease is becoming a global public health problem, which will usually cause uremia at the end stage of chronic kidney failure. So far, kidney transplant is the most effective and proper therapy for uremia, however, the short supply of matched donor kidney has been a persistent bottleneck for transplantation. HLA matching of HLA-A, -B and -DRB1 loci is very important for the allocation of kidney transplants. In this study, we investigated genotypes of HLA-A, -B and -DRB1 loci based on 1,464 uremia patients and 10,000 unrelated healthy individuals in Henan province of China, and compared the frequency distribution of these HLA alleles and corresponding haplotypes between patient and healthy groups. We detected 23 HLA-A, 49 HLA-B and 17 HLA-DRB1 alleles in total. The predominant alleles of HLA-A, -B and -DRB1 loci in patients are the same as those in healthy group. The seven most frequent alleles account for about 87%, 50%, and 77% at HLA-A, -B and -DRB1 loci, respectively. The haplotypes (combinations of HLA-A, -B, and -DRB1) with significantly different frequency between patients and controls mostly account for less than 1%. Overall, this suggests that HLA matching is not a potential difficulty for kidney transplant of uremia patients. However, three of the top seven frequent HLA-DRB1 alleles have a significantly different distribution in patients and controls, while only one alleles for HLA-B and zero for HLA-A loci. These HLA-DRB1 alleles may be closely associated with uremia. This study sheds new lights on the composition and difference of HLA genotypes in uremia patients and healthy populations in Central China that can serve as a guide to HLA matching for kidney transplants and a resource for HLA typing-related studies.

## Introduction

Chronic kidney disease (CKD) is becoming a global public health problem [1]. In China, the overall prevalence of CKD was estimated to be about 10.8% [2]. People with CKD were identified as being at high risk for cardiovascular and all-cause mortality [3], and thus CKD was considered as one of the most preventable risk factors with high mortality globally [4]. The end stage of chronic kidney failure is uremia, which will cause a series of clinical manifestations and affect nearly all the body systems [5, 6]. Without treatment, uremia due to renal failure will progress and eventually cause death. Kidney transplant is the most effective and proper therapy for uremia, though it is greatly limited to the extreme scarcity of matched kidney source.

Human leukocyte antigens (HLA), a group of genes encoding the major histocompatibility complex, have been reported to be associated with many diseases. The HLA antigens can be divided into intracellular molecules (HLA-A, -B and -C) and extracellular molecules (HLA-DPA1, -DPB1, -DQA1, -DQB1, -DRA and -DRB1) [7]. HLA are highly polymorphic, and the allele and haplotype distribution in the HLA system vary within different populations [8]. HLA matching is an important factor for the allocation of kidney transplants [9]. HLA mismatching in kidney transplantation has been shown to be associated with poorer graft and patient survival [7]. Notably, HLA-A, -B and -DRB1 antigens contribute to the majority of the immunogenicity of mismatched antigens, and therefore HLA matching for kidney transplants have primarily focused on these alleles.

In this study, we investigated the genetic polymorphisms at HLA-A, -B and -DRB1 loci in uremia patients in Henan province of China, and compared them with those in unrelated healthy individuals. A total of 23 HLA-A, 49 HLA-B and 17 HLA-DRB1 alleles were detected. Interestingly, the predominant HLA alleles and haplotypes were common in both patient and healthy groups. Besides, we also revealed some potential risky HLA genes as well as some protective genes for uremia. This finding provides useful information for allocation of kidney transplants.

## Materials and Methods

### Data collection

The study was approved by the Ethics Committee of the First Affiliated Hospital of Zhengzhou University. All the patients provided written informed consent for the study. After obtaining informed consent, blood samples were taken from 1,464 uremia patients from the First Affiliated Hospital of Zhengzhou University in Henan province. PCR-SSP (polymerase chain reaction and sequence-specific primer) typing was used to detect genotypes at the HLA-A, -B and -DRB1 loci in patients. PCR-SSP was performed using the HLA SSP ABDR Typing Kit (TBG) according to the protocol. Briefly, extracted DNA was prepared for the HLA SSP ABDR Typing Kit, and performed PCR amplification as follows: denature at 96°C for 5 min; and then 5 cycles for 20 sec at 96°C and 1 min at 68°C; followed by 10 cycles for 20 sec at 96°C, 50 sec at 64°C and 45 sec at 72°C; and further 15 cycles for 20 sec at 96°C, 50 sec at 61°C and 45 sec at 72°C; lastly, elongate at 72°C for 5 min. Finally, gel electrophoresis was performed and analyzed. As for control, HLA genotypes of 10,000 unrelated healthy individuals by PCR-SSO (polymerase chain reaction and sequence-specific oligonucleotide probe hybridization) typing were collected from the marrow donor database of Henan province in China. All of the data were from Henan Han population and were anonymous.

### Statistical analysis

Allele frequencies of HLA-A, -B, and -DRB1 loci were calculated for patient and control samples, respectively. Haplotype frequencies of HLA-A-B, HLA-A-DRB1, HLA-B-DRB1 and HLA-A-B-DRB1 were estimated by the maximum likelihood methods using the software Arlequin (version 3.5) [10].

Chi-square test was applied to test the statistical significance of the frequency difference of each HLA antigen or haplotype between uremia patients and healthy controls. For cases with expected counts less than 5, Fisher’s exact test was used instead. A corrected p-value (Pc) based on Bonferroni’s correction was used for multiple test correction, and Pc less than 0.05 were considered to be statistically significant.

## Results

### The top frequent alleles of HLA-A, -B and -DRB1 loci were common in uremia patients and healthy individuals

Based on the statistical analysis of 1,464 uremia patients and 10,000 unrelated healthy individuals, we detected 23 HLA-A alleles, 49 HLA-B alleles and 17 HLA-DRB1 alleles (S1–S3 Tables). For HLA-A and -DRB1 loci, the top seven most frequent alleles in patients were consistent with those in healthy controls (Table 1). They were A2, A11, A24, A30, A33, A3 and A1 at HLA-A locus, and DRB1*15, DRB1*4, DRB1*7, DRB1*9, DRB1*12, DRB1*11 and DRB1*14 at HLA-DRB1 locus. Notably, these predominant alleles covered more than 87% of HLA-A and 77% of HLA-DRB1 loci in both patients and controls. For HLA-B loci, the top five most frequent alleles in patients were B13, B51, B60, B46 and B61, which were almost the same as those in controls except B46 which was replaced by B62 (Table 1). These most frequent HLA-A, -B and -DRB1 alleles were also consistent with studies in Han population of China [11, 12]. Taken together, the top frequent HLA-A, -B, and -DRB1 alleles are common in patients and healthy individuals. This suggests that it is relatively easy for type matching of HLA-A and -DRB1 alleles for renal transplantation.

**Table 1:** The seven most frequent alleles of HLA-A, -B and -DRB1 loci.

Allele	Patients (1,464)		Controls (10,000)		p-value	Pc
	n	Frequency (%)	n	Frequency (%)		
**A11**	542	18.51	3294	16.47	0.006	0.145
**A1**	116	3.96	991	4.96	0.019	0.426
**A24**	480	16.39	3123	15.62	0.277	1.000
**A3**	138	4.71	1036	5.18	0.302	1.000
**A30**	235	8.03	1670	8.35	0.591	1.000
**A2**	881	30.09	5980	29.9	0.846	1.000
**A33**	196	6.69	1327	6.64	0.905	1.000
**B62**	150	5.12	1434	7.17	<0.001	0.001
**B51**	213	7.28	1590	7.95	0.211	1.000
**B35**	163	5.57	1232	6.16	0.230	1.000
**B61**	178	6.08	1334	6.67	0.248	1.000
**B46**	179	6.11	1289	6.45	0.518	1.000
**B60**	198	6.76	1294	6.47	0.547	1.000
**B13**	364	12.43	2482	12.41	0.976	1.000
DRB1*15	440	15.03	3846	19.23	<0.001	<0.001
DRB1*4	405	13.83	2167	10.84	<0.001	<0.001
DRB1*11	260	8.88	1291	6.46	<0.001	<0.001
DRB1*9	330	11.27	2571	12.86	0.016	0.270
DRB1*12	317	10.83	1992	9.96	0.148	1.000
DRB1*14	179	6.11	1132	5.66	0.327	1.000
DRB1*7	361	12.33	2481	12.41	0.928	1.000

### Potential risky alleles of HLA-A, -B and -DRB1 loci for uremia patients

**Fig 1:**
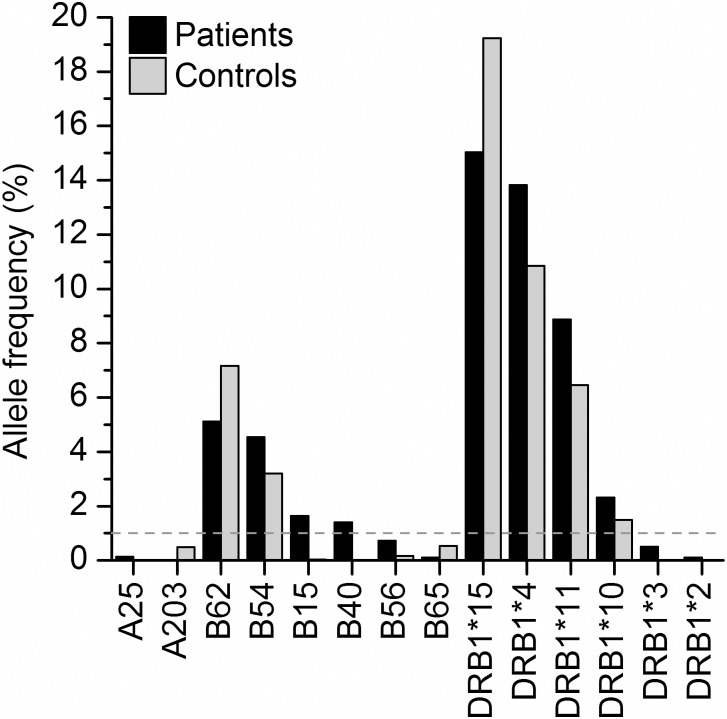
HLA-A, -B and -DRB1 alleles with significantly different frequencies between uremia patients and healthy controls.

We examined the statistic significance of the frequency difference for individual HLA alleles between uremia patients and healthy controls. Of the 23 HLA-A alleles, only two alleles (A25 and A203) show significantly different frequencies in patient and healthy populations (Fig 1), and both of them are rarely observed (S1 Table). Of the 49 HLA-B alleles, six alleles (B62, B54, B15, B40, B56, and B65) show significantly different frequencies in patients and healthy groups (Fig 1), and four of them (B62, B54, B15, and B40) show frequencies greater than 1% in uremia patients (Fig 1). Of the 17 HLA-DRB1 alleles, six alleles (DRB1*15, DRB1*4, DRB1*11, DRB1*10, DRB1*3, and DRB1*2) show significantly different frequencies in uremia patients. Notably, four of them (DRB1*15, DRB1*4, DRB1*11, and DRB1*10) show frequencies greater than 1% in uremia patients (Fig 1), and the total frequency of these four alleles is 40.06% (S3 Table). Of the significantly distributed alleles with frequency greater than 1%, six alleles (B54, B15, B40, DRB1*4, DRB1*11, and DRB1*10) show significantly higher frequencies in uremia patients, and two of the six (DRB1*4 and DRB1*11) belong to the top seven frequent alleles which may be risky alleles for uremia. The results showed that individual alleles at HLA-DRB1 loci exhibited most unbalanced distribution between patients and healthy population. HLA-DRB1 alleles seems to be more closely associated with uremia patients and should be more weighted in HLA matching during allocation of kidney transplants.

### Similar distribution of HLA haplotype frequencies in uremia patients and healthy individuals

We estimated frequencies of two and three loci haplotypes at HLA-A, HLA-B and HLA-DRB1 loci in the patient group using maximum likelihood method [10], and compared them with those in the control group.

We detected total 470 HLA-A-B haplotypes, 229 HLA-A-DRB1 haplotypes, 468 HLA-B-DRB1 haplotypes, and 2186 HLA-A-B-DRB1 haplotypes. More than half of the haplotypes have a frequency of less than 2%. Of the 10 most frequent haplotypes of these four combinations each, only two haplotypes (A2-DRB1*15 and A11-DRB1*4) have significantly different frequencies between patients and controls (Table 2). There are in total 31 haplotypes with significantly different frequencies between patients and controls (Table 3), and only four of them (A24-B54, A2-DRB1*15, A11-DRB1*4, and A24-DRB1*7) have a frequency of greater than 1% in patients. This suggests that haplotypes have a similar distribution in patients and healthy population.

**Table 2:** The 10 most frequent haplotypes of HLA-A-B, HLA-A-DRB1, HLA-B-DRB1 and HLA-A-B-DRB1.

Haplotype	HF^1^ in patients (%)	HF in controls (%)	Haplotype	HF in patients (%)	HF in controls (%)
**A30-B13**	6.319	7.133	B13-DRB1*7	6.637	6.816
**A2-B46**	4.015	4.558	B46-DRB1*9	2.428	2.801
**A2-B61**	3.161	2.831	B54-DRB1*4	2.311	1.430
**A11-B60**	3.037	2.176	B13-DRB1*12	1.903	2.405
**A33-B58**	2.949	2.672	B58-DRB1*17	1.938	1.558
**A2-B51**	2.598	2.560	B52-DRB1*15	1.693	2.241
**A2-B13**	2.394	2.036	B62-DRB1*4	1.661	1.688
**A2-B62**	1.589	2.442	B61-DRB1*9	1.425	1.865
**A11-B62**	1.801	1.947	B44-DRB1*7	1.388	1.764
**A2-B75**	1.331	2.206	B7-DRB1*15	1.364	2.138
A30-DRB1*7	5.245	5.668	A30-B13-DRB1*7	5.189	5.593
A2-DRB1*9	4.877	5.620	A2-B46-DRB1*9	1.912	1.961
A2-DRB1*4	4.476	3.147	A33-B58-DRB1*17	1.302	1.145
A2-DRB1*15	4.408	6.216	A2-B13-DRB1*12	1.245	1.265
A2-DRB1*12	4.322	3.994	A1-B37-DRB1*10	1.086	0.780
A11-DRB1*4	3.800	2.481	A11-B62-DRB1*4	0.975	0.698
A11-DRB1*9	2.850	2.261	A2-B61-DRB1*9	0.972	0.675
A11-DRB1*12	2.794	2.584	A24-B54-DRB1*4	0.953	0.607
A24-DRB1*15	2.622	3.490	A33-B44-DRB1*13	0.769	0.978
A11-DRB1*15	2.590	3.759	A3-B7-DRB1*15	0.675	0.782

^1^ HF: haplotype frequency

**Table 3:** The haplotypes with significantly different frequencies between uremia patients and healthy controls.

Haplotype	HF^1^ in patients (%)	HF in controls (%)	p-value	Pc
**A2-B15**	0.666	0.000	<0.001	<0.001
**A24-B40**	0.491	0.000	<0.001	<0.001
**A11-B15**	0.435	0.000	<0.001	<0.001
**A2-B40**	0.291	0.000	<0.001	<0.001
**A11-B40**	0.253	0.000	<0.001	<0.001
**A24-B15**	0.197	0.000	<0.001	0.002
**A2-B56**	0.382	0.064	<0.001	0.009
**A26-B54**	0.294	0.029	<0.001	0.010
**A24-B54**	2.207	1.240	<0.001	0.036
A24-DRB1*7	1.467	0.573	<0.001	<0.001
A33-DRB1*3	0.164	0.000	<0.001	0.008
A11-DRB1*4	3.800	2.481	<0.001	0.014
A2-DRB1*15	4.408	6.216	<0.001	0.024
B15-DRB1*11	0.583	0.000	<0.001	<0.001
B40-DRB1*15	0.374	0.000	<0.001	<0.001
B40-DRB1*12	0.230	0.000	<0.001	<0.001
B15-DRB1*4	0.212	0.000	<0.001	<0.001
B15-DRB1*9	0.209	0.000	<0.001	<0.001
B62-DRB1*15	0.939	2.198	<0.001	0.001
B15-DRB1*15	0.343	0.032	<0.001	0.001
B40-DRB1*9	0.202	0.000	<0.001	0.002
B40-DRB1*11	0.173	0.000	<0.001	0.002
B58-DRB1*3	0.165	0.000	<0.001	0.016
B40-DRB1*4	0.153	0.000	<0.001	0.016
A2-B15-DRB1*11	0.408	0.000	<0.001	<0.001
A26-B35-DRB1*15	0.264	0.000	<0.001	<0.001
A2-B40-DRB1*15	0.208	0.000	<0.001	0.001
A2-B55-DRB1*9	0.204	0.000	<0.001	0.009
A11-B15-DRB1*15	0.195	0.000	<0.001	0.009
A24-B54-DRB1*8	0.283	0.022	<0.001	0.021
A24-B35-DRB1*15	0.000	0.425	<0.001	0.047

^1^ HF: haplotype frequency

Given that A2, A11, A24, A30, A33, B13, B51, B60, B61, B46, DRB1*15, DRB1*7, DRB1*4, DRB1*9 and DRB1*12 are most frequent in both patients and controls, it’s expected that the haplotypes consisted of these alleles also had the highest frequencies. Patients with those HLA genes are thought to be relatively easy to find matched donors.

## Discussion

In this study, genotypes of HLA-A, HLA-B and HLA-DRB1 loci were collected from 1,464 uremia patients and 10,000 unrelated healthy individuals in Henan province of China. A total of 23 HLA-A alleles, 49 HLA-B alleles and 17 HLA-DRB1 alleles have been detected, and the top frequent alleles of HLA-A, -B and -DRB1 loci in patients are nearly the same as those in healthy individuals (Table 1), suggesting the convenience for allocation of kidney transplantation. However, some alleles show significantly different frequencies between patients and controls. For alleles with frequencies of greater than 1% each in patients, two of them (B62 and DRB1*15) are significantly enriched in healthy individuals (Fig 1), which may be protective alleles for uremia. Notably, six alleles (DRB1*4, DRB1*11, DRB1*10, B54, B15, and B40) are significantly more frequent in patients (Fig 1), suggesting that they may be risky alleles for uremia and may increase the difficulty in allocation of kidney transplantation, thus these alleles should be paid more attention.

For haplotypes of two or three HLA loci, we found the most frequent haplotypes were quite common in patients and controls (Table 2). Of the 10 most frequent haplotypes of these four combinations each, only A2-DRB1*15 was significantly less frequent in patients, and only A11-DRB1*4 was significantly more frequent in patients than that in controls (Tables 2 and 3). This suggests that haplotypes have a similar distribution in patients and healthy population.

Since the HLA alleles are highly polymorphic and vary within different populations, previous studies investigated the frequency distribution of HLA alleles in different ethnic populations. Papasteriades et al [13] reported that patients with microscopic polyarteritis and renal involvement in Greek had increased frequency of HLA-A26 and A11 and decreased frequency of DRB1*3. Freedman et al [14] reported that HLA-DRB1*3 and DRB1*5 are positively associated with end-stage renal disease (ESRD) due to membranous glomerulonephritis in American patients. Karahan et al [8] reported that the frequency of HLA-B58 and DRB1*3 was significantly higher in Turkish ESRD patients. Crispim et al [15] reported that HLA-A78 and DRB1*11 were significantly more frequent in Brazilian ESRD patients and the antigens HLA-B14 was present at a significantly lower frequency in patients compared with controls. Spriewald et al [16] reported that there was no significant difference between Wegener’s granulomatosis-caused ESRD patients and normal people in Germany. EI-Gezawy et al [17] reported that HLA-A2, DRB1*3 and DRB1*11 had significantly higher frequency in Egyptian ESRD patients and HLA-B8 had significantly lower frequency. Given above, the higher frequencies of HLA-DRB1*3 and DRB1*11 in patients in our study were consistent with the studies in America, Turkey, Brazil and Egypt. However, there were still many differences among these studies, which may be due to the diversity of of HLA alleles in different populations and areas.

Besides polymorphism of HLA alleles in different ethnic populations [18], we wondered the haplotype distribution of same population in different areas. As the study of HLA-A-B haplotypes in Hongkong reported, the frequencies of A30-B13, A2-B46, A33-B58 and A11-B60 were 1.34%, 10.10%, 5.36% and 6.36% respectively [19], which were quite different from our results (Table 2). For HLA-A-B-DRB1 haplotypes, it was reported that the frequencies of A30-B13-DRB1*7, A2-B46-DRB1*9, A33-B58-DRB1*17 and A1-B37-DRB1*10 were 3.39%, 0.32%, 1.63% and 0.87% respectively in Xi’an city of China [11]. The first two haplotype frequencies were quite different from our results (Table 2). It was also reported that in Taiwan Han population, the frequencies of A30-B13, A2-B46, A33-B58 and A11-B60 was 1.09%, 10.12%, 8.94% and 8.91% respectively, while the frequencies of B13-DRB1*7, B46-DRB1*9, B13-DRB1*12, A30-B13-DRB1*7 and A2-B46-DRB1*9 were 1.28%, 6.68%, 1.58%, 0.94% and 4.72% respectively [20]. Therefore, the HLA genotypes were quite diverse even in different areas of the same ethnic population.

Due to the high polymorphism of HLA alleles and haplotypes in different populations and even in different areas of the same population, together with the importance of HLA matching for the allocation of kidney transplants, reliable statistics of HLA genotypes in each population is essential to estimate the probability of finding compatible donors for uremia patients. Comparison analysis of HLA alleles and haplotypes between uremia patients and healthy individuals in this study will greatly benefit renal transplantation in Henan province and neighboring regions in China, and also provide useful information for HLA-related studies.

## Supporting Information

S1 Table Frequency distribution of HLA-A alleles. (DOC)

S2 Table Frequency distribution of HLA-B alleles. (DOC)

S3 Table Frequency distribution of HLA-DRB1 alleles. (DOC)
