# Supplementary material for: Comparison of HLA-A, -B and -DRB1 Loci Polymorphism between Kidney Transplants of Uremia Patients and Healthy Individuals in Central China
Source: PLoS One. 2016 Oct 25;11(10):e0165426. doi: 10.1371/journal.pone.0165426 (PMC5079547; doi:10.1371/journal.pone.0165426)
Supplement: S1 Table — (DOC) [file pone.0165426.s001.doc]

S1 Table. Frequency distribution of HLA-A alleles.

| **Allele** | **Patients (1,464)** | | **Controls (10,000)** | | **p-value** | **Pc** |
| --- | --- | --- | --- | --- | --- | --- |
|  | **n** | **Frequency (%)** | **n** | **Frequency (%)** |  |  |
| **A2** | 881 | 30.09 | 5980 | 29.90 | 0.846 | 1.000 |
| **A11** | 542 | 18.51 | 3294 | 16.47 | 0.006 | 0.145 |
| **A24** | 480 | 16.39 | 3123 | 15.62 | 0.277 | 1.000 |
| **A30** | 235 | 8.03 | 1670 | 8.35 | 0.591 | 1.000 |
| **A33** | 196 | 6.69 | 1327 | 6.64 | 0.905 | 1.000 |
| **A3** | 138 | 4.71 | 1036 | 5.18 | 0.302 | 1.000 |
| **A1** | 116 | 3.96 | 991 | 4.96 | 0.019 | 0.426 |
| **A31** | 111 | 3.79 | 807 | 4.04 | 0.579 | 1.000 |
| **A26** | 99 | 3.38 | 600 | 3.00 | 0.274 | 1.000 |
| **A32** | 52 | 1.78 | 476 | 2.38 | 0.041 | 0.939 |
| **A68** | 35 | 1.20 | 222 | 1.11 | 0.639 | 1.000 |
| **A29** | 22 | 0.75 | 247 | 1.24 | 0.021 | 0.491 |
| **A23** | 11 | 0.38 | 82 | 0.41 | 0.877 | 1.000 |
| **A203 ^#^** | 0 | 0.00 | 98 | 0.49 | <0.001 | <0.001 |
| **A69** | 3 | 0.10 | 13 | 0.07 | 0.448 | 1.000 |
| **A25 ^#^** | 4 | 0.14 | 1 | 0.01 | 0.001 | 0.027 |
| **A66** | 0 | 0.00 | 16 | 0.08 | 0.250 | 1.000 |
| **A34** | 0 | 0.00 | 9 | 0.05 | 0.615 | 1.000 |
| **A28** | 1 | 0.03 | 0 | 0.00 | 0.128 | 1.000 |
| **A36** | 1 | 0.03 | 0 | 0.00 | 0.128 | 1.000 |
| **A43** | 1 | 0.03 | 0 | 0.00 | 0.128 | 1.000 |
| **A210** | 0 | 0.00 | 5 | 0.03 | 1.000 | 1.000 |
| **A74** | 0 | 0.00 | 3 | 0.02 | 1.000 | 1.000 |

**^#^**Pc < 0.05
