# Supplementary material for: Comparison of HLA-A, -B and -DRB1 Loci Polymorphism between Kidney Transplants of Uremia Patients and Healthy Individuals in Central China
Source: PLoS One. 2016 Oct 25;11(10):e0165426. doi: 10.1371/journal.pone.0165426 (PMC5079547; doi:10.1371/journal.pone.0165426)
Supplement: S2 Table — (DOC) [file pone.0165426.s002.doc]

S2 Table. Frequency distribution of HLA-B alleles.

| **Allele** | **Patients (1,464)** | | **Controls (10,000)** | | **p-value** | **Pc** |
| --- | --- | --- | --- | --- | --- | --- |
|  | **n** | **Frequency (%)** | **n** | **Frequency (%)** |  |  |
| **B13** | 364 | 12.43 | 2482 | 12.41 | 0.976 | 1.000 |
| **B51** | 213 | 7.28 | 1590 | 7.95 | 0.211 | 1.000 |
| **B60** | 198 | 6.76 | 1294 | 6.47 | 0.547 | 1.000 |
| **B61** | 178 | 6.08 | 1334 | 6.67 | 0.248 | 1.000 |
| **B46** | 179 | 6.11 | 1289 | 6.45 | 0.518 | 1.000 |
| **B62 ^#^** | 150 | 5.12 | 1434 | 7.17 | <0.001 | 0.001 |
| **B35** | 163 | 5.57 | 1232 | 6.16 | 0.230 | 1.000 |
| **B44** | 126 | 4.30 | 966 | 4.83 | 0.227 | 1.000 |
| **B7** | 105 | 3.59 | 934 | 4.67 | 0.008 | 0.376 |
| **B58** | 126 | 4.30 | 734 | 3.67 | 0.096 | 1.000 |
| **B75** | 112 | 3.83 | 815 | 4.08 | 0.547 | 1.000 |
| **B54 ^#^** | 133 | 4.54 | 640 | 3.20 | <0.001 | 0.014 |
| **B48** | 102 | 3.48 | 723 | 3.62 | 0.750 | 1.000 |
| **B52** | 94 | 3.21 | 698 | 3.49 | 0.481 | 1.000 |
| **B38** | 71 | 2.43 | 571 | 2.86 | 0.208 | 1.000 |
| **B27** | 81 | 2.77 | 416 | 2.08 | 0.021 | 1.000 |
| **B55** | 76 | 2.60 | 354 | 1.77 | 0.003 | 0.167 |
| **B37** | 60 | 2.05 | 300 | 1.50 | 0.031 | 1.000 |
| **B71** | 43 | 1.47 | 356 | 1.78 | 0.256 | 1.000 |
| **B39** | 47 | 1.61 | 325 | 1.63 | 1.000 | 1.000 |
| **B57** | 35 | 1.20 | 273 | 1.37 | 0.493 | 1.000 |
| **B8** | 32 | 1.09 | 250 | 1.25 | 0.530 | 1.000 |
| **B67** | 27 | 0.92 | 217 | 1.09 | 0.499 | 1.000 |
| **B15 ^#^** | 48 | 1.64 | 7 | 0.04 | <0.001 | <0.001 |
| **B50** | 26 | 0.89 | 152 | 0.76 | 0.431 | 1.000 |
| **B40 ^#^** | 41 | 1.40 | 0 | 0.00 | <0.001 | <0.001 |
| **B18** | 16 | 0.55 | 119 | 0.60 | 0.897 | 1.000 |
| **B56 ^#^** | 21 | 0.72 | 31 | 0.16 | <0.001 | <0.001 |
| **B65 ^#^** | 3 | 0.10 | 105 | 0.53 | 0.001 | 0.034 |
| **B49** | 8 | 0.27 | 65 | 0.33 | 0.860 | 1.000 |
| **B45** | 8 | 0.27 | 46 | 0.23 | 0.681 | 1.000 |
| **B63** | 6 | 0.21 | 43 | 0.22 | 1.000 | 1.000 |
| **B81** | 5 | 0.17 | 26 | 0.13 | 0.586 | 1.000 |
| **B41** | 4 | 0.14 | 28 | 0.14 | 1.000 | 1.000 |
| **B72** | 3 | 0.10 | 26 | 0.13 | 1.000 | 1.000 |
| **B76** | 5 | 0.17 | 12 | 0.06 | 0.056 | 1.000 |
| **B53 ^#^** | 4 | 0.14 | 4 | 0.02 | 0.012 | 0.594 |
| **B42 ^#^** | 4 | 0.14 | 1 | 0.01 | 0.001 | 0.058 |
| **B5102 ^#^** | 0 | 0.00 | 28 | 0.14 | 0.042 | 1.000 |
| **B59** | 1 | 0.03 | 20 | 0.10 | 0.508 | 1.000 |
| **B64** | 1 | 0.03 | 19 | 0.10 | 0.502 | 1.000 |
| **B47** | 1 | 0.03 | 14 | 0.07 | 0.710 | 1.000 |
| **B77** | 2 | 0.07 | 5 | 0.03 | 0.222 | 1.000 |
| **B70** | 2 | 0.07 | 4 | 0.02 | 0.172 | 1.000 |
| **B22** | 2 | 0.07 | 3 | 0.02 | 0.125 | 1.000 |
| **B3901** | 0 | 0.00 | 12 | 0.06 | 0.384 | 1.000 |
| **B14** | 1 | 0.03 | 1 | 0.01 | 0.239 | 1.000 |
| **B78** | 1 | 0.03 | 0 | 0.00 | 0.128 | 1.000 |
| **B73** | 0 | 0.00 | 2 | 0.01 | 1.000 | 1.000 |

**^#^** Pc < 0.05
