# Supplementary material for: Comparison of HLA-A, -B and -DRB1 Loci Polymorphism between Kidney Transplants of Uremia Patients and Healthy Individuals in Central China
Source: PLoS One. 2016 Oct 25;11(10):e0165426. doi: 10.1371/journal.pone.0165426 (PMC5079547; doi:10.1371/journal.pone.0165426)
Supplement: S3 Table — (DOC) [file pone.0165426.s003.doc]

S3 Table. Frequency distribution of HLA-DRB1 alleles.

| **Allele** | **Patients (1,464)** | | **Controls (10,000)** | | **p-value** | **Pc** |
| --- | --- | --- | --- | --- | --- | --- |
|  | **n** | **Frequency (%)** | **n** | **Frequency (%)** |  |  |
| **DRB1*15 ^#^** | 440 | 15.03 | 3846 | 19.23 | <0.001 | <0.001 |
| **DRB1*7** | 361 | 12.33 | 2481 | 12.41 | 0.928 | 1.000 |
| **DRB1*4 ^#^** | 405 | 13.83 | 2167 | 10.84 | <0.001 | <0.001 |
| **DRB1*9** | 330 | 11.27 | 2571 | 12.86 | 0.016 | 0.270 |
| **DRB1*12** | 317 | 10.83 | 1992 | 9.96 | 0.148 | 1.000 |
| **DRB1*11 ^#^** | 260 | 8.88 | 1291 | 6.46 | <0.001 | <0.001 |
| **DRB1*14** | 179 | 6.11 | 1132 | 5.66 | 0.327 | 1.000 |
| **DRB1*8** | 149 | 5.09 | 1109 | 5.55 | 0.339 | 1.000 |
| **DRB1*13** | 151 | 5.16 | 1004 | 5.02 | 0.751 | 1.000 |
| **DRB1*17** | 102 | 3.48 | 741 | 3.71 | 0.599 | 1.000 |
| **DRB1*1** | 81 | 2.77 | 719 | 3.60 | 0.023 | 0.397 |
| **DRB1*16** | 67 | 2.29 | 626 | 3.13 | 0.013 | 0.218 |
| **DRB1*10 ^#^** | 68 | 2.32 | 298 | 1.49 | 0.002 | 0.026 |
| **DRB1*3 ^#^** | 15 | 0.51 | 1 | 0.01 | <0.001 | <0.001 |
| **DRB1*2 ^#^** | 3 | 0.10 | 0 | 0.00 | 0.002 | 0.035 |
| **DRB1*1403** | 0 | 0.00 | 12 | 0.06 | 0.384 | 1.000 |
| **DRB1*1404** | 0 | 0.00 | 10 | 0.05 | 0.627 | 1.000 |

**^#^** Pc < 0.05
